# Effects of autophagy-inhibiting chemicals on sialylation of Fc-fusion glycoprotein in recombinant CHO cells

**DOI:** 10.1007/s00253-024-13059-9

**Published:** 2024-02-20

**Authors:** Hoon-Min Lee, Jong-Ho Park, Tae-Ho Kim, Hyun-Seung Kim, Dae Eung Kim, Mi Kyeong Lee, Jungmok You, Gyun Min Lee, Yeon-Gu Kim

**Affiliations:** 1 Biotherapeutics Translational Research Center, Korea Research Institute of Bioscience and Biotechnology (KRIBB), 125 Gwahak-ro, Yuseong-gu, Daejeon, Korea; 2 Department of Bioprocess Engineering, KRIBB School of Biotechnology, University of Science and Technology (UST), 217 Gajeong-ro, Yuseong-gu, Daejeon, Korea; 3 Department of Biological Sciences, KAIST, 335 Gwahak-ro, Yuseong-gu, Daejeon, Korea; 4 Department of Plant and Environmental New Resources, Graduate School of Biotechnology, College of Life Science, Kyung Hee University, 1732 Deogyeong-daero, Giheung-gu, Yongin-si, Gyeonggi-do Korea; 5 College of Pharmacy, Chungbuk National University, Cheongju, 28160 Korea

**Keywords:** Autophagy, Recombinant CHO cells, Fc-fusion glycoprotein production, Sialylation, Nucleotide sugars

## Abstract

**Abstract:**

The occurrence of autophagy in recombinant Chinese hamster ovary (rCHO) cell culture has attracted attention due to its effects on therapeutic protein production. Given the significance of glycosylation in therapeutic proteins, this study examined the effects of autophagy-inhibiting chemicals on sialylation of Fc-fusion glycoproteins in rCHO cells. Three chemical autophagy inhibitors known to inhibit different stages were separately treated with two rCHO cell lines that produce the same Fc-fusion glycoprotein derived from DUKX-B11 and DG44. All autophagy inhibitors significantly decreased the sialylation of Fc-fusion glycoprotein in both cell lines. The decrease in sialylation of Fc-fusion glycoprotein is unlikely to be attributed to the release of intracellular enzymes, given the high cell viability and low activity of extracellular sialidases. Interestingly, the five intracellular nucleotide sugars remained abundant in cells treated with autophagy inhibitors. In the mRNA expression profiles of 27 *N*-glycosylation-related genes using the NanoString nCounter system, no significant differences in gene expression were noted. With the positive effect of supplementing nucleotide sugar precursors on sialylation, attempts were made to enhance the levels of intracellular nucleotide sugars by supplying these precursors. The addition of nucleotide sugar precursors to cultures treated with inhibitors successfully enhanced the sialylation of Fc-fusion glycoproteins compared to the control culture. This was particularly evident under mild stress conditions and not under relatively severe stress conditions, which were characterized by a high decrease in sialylation. These results suggest that inhibiting autophagy in rCHO cell culture decreases sialylation of Fc-fusion glycoprotein by constraining the availability of intracellular nucleotide sugars.

**Key points:**

*•  The autophagy inhibition in rCHO cell culture leads to a significant reduction in the sialylation of Fc-fusion glycoprotein.*

*•  The pool of five intracellular nucleotide sugars remained highly abundant in cells treated with autophagy inhibitors.*

*•  Supplementation of nucleotide sugar precursors effectively restores decreased sialylation, particularly under mild stress conditions but not in relatively severe stress conditions.*

**Graphical Abstract:**

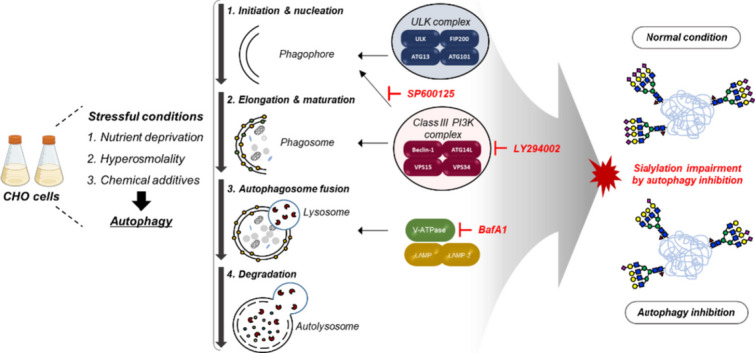

**Supplementary Information:**

The online version contains supplementary material available at 10.1007/s00253-024-13059-9.

## Introduction

In the biopharmaceutical industry, recombinant Chinese hamster ovary (rCHO) cells are the most commonly used mammalian cell line to efficiently produce therapeutic glycoproteins due to their ability to produce human-like glycosylation patterns. Glycosylation is considered one of the critical quality attributes of glycoproteins because it can significantly impact the efficacy of therapeutic proteins (Hossler et al. 2009). In particular, sialylation of *N*-glycosylation plays a crucial role in the biological functions of protein, such as immunogenicity, stability, and *in vivo* half-life (Elliott et al. 2003). Therefore, it is important to comprehend the factors that can influence glycosylation in therapeutic glycoprotein production during rCHO cell culture to achieve the desired protein quality.

Impairment of glycosylation in rCHO cell culture occurs under various stressful culture conditions, including nutrient deprivation, byproduct accumulation, chemical additives, and hyperosmolality (Ha et al. 2022). The stressful conditions ultimately lead to cell death, during which the glycosylation of extracellular proteins is impaired due to the release of intracellular as proteases and glycosidases with membrane disruption (Sung et al. 2004). Therefore, numerous comprehensive studies have been conducted in rCHO cell culture to mitigate apoptosis, a type I programmed cell death (PCD), aiming to prevent sialylation damage to recombinant glycoproteins. This has been achieved through genetic engineering strategies such as anti-apoptosis genes and/or cell line-specific feeding strategies for fed-batch culture (Arden and Betenbaugh 2004). Furthermore, protein glycosylation can be affected by altering the expression levels of *N*-glycosylation-related genes, such as glycosyltransferases and nucleotide sugar synthesis, or by changing the substrate concentration for intracellular nucleotide sugar precursors (Ha et al. 2022). Attempts to restore impaired *N*-glycosylation-related genes using engineered CHO cells and/or supplementation of specific precursors were made to enhance protein glycosylation (Bork et al. 2009; Tejwani et al. 2018).

Autophagy is a recycling process that self-cannibalizes intracellular components through lysosomal degradation and uses them as substrates for biosynthesis and energy production (Rabinowitz and White 2010). Autophagic cell death, also known as type II PCD, exhibits distinct morphological features from apoptosis and is induced by excessive levels of cellular autophagy. However, it can function as a pro-survival mechanism under mild stress conditions (Levine and Yuan 2005). In rCHO cell culture, autophagy has been observed at the end of the batch culture under nutrient deprivation conditions (Hwang and Lee 2008), and it is also induced by hyperosmolality and the addition of sodium butyrate during batch culture (Han et al. 2010; Lee and Lee 2012a). Nutrient supply during fed-batch culture for rCHO cells inhibits or delays autophagy compared to batch culture (Han et al. 2011). CHO cells genetically engineered to overexpress anti-apoptotic proteins can delay autophagic cell death during nutrient deprivation, thereby enhancing cell viability and extending culture longevity (Kim et al. 2009).

As autophagy frequently occurs in rCHO cell culture, studies examining the effects of autophagy on cell growth and protein productivity in rCHO cells have been conducted using pharmacological autophagy inducers or inhibitors. Cultures with chemical autophagy inducer or inhibitor in antibody-producing rCHO cells have shown that autophagy induction is crucial for the cell survival under nutrient depletion conditions at the end of culture (Han et al. 2011). The addition of rapamycin, a typical autophagy inducer, increases antibody production in rCHO cells by extending culture longevity without increasing specific protein productivity (*q*_p_) (Lee and Lee 2012b). The positive effect of autophagy inhibition on *q*_p_ varies significantly based on the cell line, as evidenced by the treatment of nine chemical autophagy inhibitors known to inhibit three stages of autophagy in three rCHO cell lines (Baek et al. 2016). However, despite the increasing importance of glycosylation in protein quality, there have been no attempts to investigate the relationship between autophagy and sialylation in CHO cell culture.

In this study, we investigated the effects of autophagy-inhibiting chemicals on the sialylation of Fc-fusion glycoprotein in rCHO cells using three distinct chemicals that inhibit different stages of autophagy. The proportion of the isoform distribution and sialylated *N*-glycan of the Fc-fusion glycoprotein in this study was analyzed through isoelectric focusing (IEF) and anion exchange liquid chromatography, respectively. Additionally, we analyzed the levels of intracellular nucleotide sugars through ion-pair reversed-phase HPLC analysis and assessed the expression levels of *N*-glycosylation-related genes using the NanoString nCounter system. These analyses were conducted to achieve a comprehensive understanding of the intracellular events relevant.

## Materials and methods

### Cell line and culture maintenance

Two rCHO cell lines, namely DUKX-Fc cells and DG44-Fc cells, were used in this study. The stable DUKX-Fc cells and DG44-Fc cells, derived from DUKX-B11 and DG44, respectively, were engineered to produce the same Fc-fusion glycoprotein (Lee et al. 2015). Both cell lines were grown in serum-free suspension culture mode in 125-mL Erlenmeyer flasks (Corning, Corning, NY, USA) containing 30 mL of SFM4CHO (Hyclone, Little Chalfont, UK) supplemented with 4 mM glutamine. The two cell lines were cultured in a Climo-shaking CO_2_ incubator (Adolf Kühner, Birsfelden, Switzerland) at 37 °C, 110 rpm, and 70% humidity.

### Cell culture and chemical treatment

Cells in exponential growth phase were inoculated at a concentration of 2.0 × 10^6^ cells/mL into 125-mL Erlenmeyer flasks, each containing 30 mL of SFM4CHO with 4 mM glutamine.

The autophagy inhibitors, SP600125, LY294002, and bafilomycin A1 (BafA1) (InvivoGen, San Diego, CA, USA) were prepared by dissolving them in dimethyl sulfoxide (DMSO) and were individually added to the culture at a concentration of 30 μM, 20 μM, and 5 nM, respectively, on day 0. Cell culture without any chemical addition was used as a control, and the same volume of solvent (DMSO) was used as a vehicle control.

The nucleotide sugar precursors, *N*-acetylmannosamine (ManNAc), galactose (Gal), glucose (Glc), glucosamine (GlcN), and *N*-acetylglucosamine (GlcNAc) (Sigma-Aldrich, St. Louis, MO, USA), were added to the cultures at concentrations of 20, 10, 10, 10, and 10 mM, respectively, on day 0. Specifically, ManNAc, Gal, and GlcNAc were combined as a mixture of nucleotide sugar precursors, and this mixture was treated with autophagy inhibitors on day 0.

Cell culture samples were taken daily from the flasks to determine the viable cell density, viability, and Fc-fusion glycoprotein concentration and were kept at −70 °C for further analysis.

### Cell density, viability, and Fc-fusion glycoprotein assay

Cell density and viability were assessed using a Cedex HiRes analyzer (Roche Diagnostics, Basel, Switzerland) employing the trypan blue dye exclusion method. The concentration of secreted Fc-fusion glycoprotein in the culture supernatant was determined using a Cedex Bio analyzer (Roche Diagnostics) following the manufacturer’s instructions.

### Purification of Fc-fusion glycoprotein and IEF gel analysis

The Fc-fusion glycoprotein from cell culture supernatants was purified by protein A chromatography with HiTrap™ Fibro PrismA (Cytiva, Little Chalfont, UK) and a liquid chromatography (LC) system (ÄKTA pure 25 L; Cytiva). Equal amounts of purified Fc-fusion glycoprotein were then electrophoresed on Novex™ pH 3-7 IEF Protein Gels (Thermo Fisher Scientific, Waltham, MA, USA), and the gels were stained with InstantBlue™ (Expedeon, Cambridge, UK) following the manufacturer’s instructions.

### Analysis of sialic acid content

The quantification of sialic acid content in the purified Fc-fusion glycoproteins was performed through an EnzyChrom Sialic Acid Assay Kit (BioAssay Systems, Hayward, CA, USA) and a Sialic Acid Quantitation Kit (Agilent Technologies, Santa Clara, CA, USA).

### Analysis of sialylated *N*-glycans of the Fc-fusion glycoprotein

The analysis of sialylated *N*-glycans in the purified Fc-fusion glycoprotein was conducted as previously described (Lee et al. 2017). In brief, the *N*-glycans of Fc-fusion glycoprotein were enzymatically released by PNGase F (Roche Diagnostics). The released *N*-glycans were then lyophilized, labeled with 2-aminobenzamide (2-AB) solution (Sigma-Aldrich), and subsequently injected directly into an Agilent Bio SAX column (5 μm, 2.1 mm × 250 mm; Agilent Technologies) connected to an ultra-high-performance liquid chromatography (UHPLC) system (1290 Infinity II Bio LC system; Agilent Technologies). The *N*-glycans labeled with 2-AB were observed through a fluorescence detector with an excitation wavelength of 260 nm and an emission wavelength of 430 nm.

### Analysis of sialidase and sialyltransferase activity

The enzyme activities of intracellular/extracellular sialidase, as well as intracellular α2,3-sialyltransferase (α2,3-ST), were measured by the Amplex Red Neuraminidase Assay Kit (Thermo Fisher Scientific) and the Sialyltransferase Activity Kit (R&D Systems, MN, USA), respectively, following the manufacturer’s instructions. The intracellular activities of both sialidase and sialyltransferase were measured using cell lysates from 1 × 10^6^ cells. The extracellular sialidase activity was determined from cell culture supernatants.

### Quantification of intracellular nucleotide sugars by ion-pair reversed-phase HPLC analysis

The intracellular nucleotide sugars were extracted from cell culture samples as previously described (Kochanowski et al. 2006). Briefly, 3.0 × 10^6^ cells were harvested and resuspended in ice-cold 500 mM perchloric acid (Sigma-Aldrich). The mixture was incubated on ice for 2 min and then centrifuged at 18,000 g at 4 °C for 5 min. The supernatant was neutralized using ice-cold 2.5 M KOH (Sigma-Aldrich) in 1.1 M K_2_HPO_4_ (Sigma-Aldrich). The neutralized sample was centrifuged and stored at −70 °C for further UHPLC analysis.

The separation and quantification of intracellular nucleotide sugars were carried out using ion-pair reversed-phase chromatography as described previously (Kochanowski et al. 2006). Nucleotide sugars were identified based on their absorbance at 254 nm, employing a Waters ACQUITY Premier HSS T3 column with VanGuard FIT (1.8 μm, 2.1 mm × 150 mm; Waters, Milford, MA, USA) connected to an UHPLC system (1290 Infinity II Bio LC system; Agilent Technologies). Mobile phase A consisted of 100 mM potassium phosphate (Sigma-Aldrich), pH 6.5 supplemented with 8 mM tetrabutylammonium bisulfate (Sigma-Aldrich) as an ion-pair reagent, while mobile phase B comprised 70% of mobile phase A and 30% acetonitrile. The flow rate and temperature were set to 0.4 mL/min and 40 °C, respectively.

Peak identification and integration of nucleotide sugars were achieved using a mixture of standards. The standard mixture contained five nucleotide sugars: CMP-sialic acid (CMP-SA), UDP-galactose (UDP-Gal), UDP-glucose (UDP-Glc), UDP-GlcNAc, and GDP-fucose (GDP-Fuc). The integrated peak areas of the standard mixture were calculated using OpenLab CDS ChemStation software (Agilent Technologies) following UHPLC analysis.

### Gene expression level analysis using NanoString nCounter system

The analysis of gene expression level was conducted using NanoString nCounter system (Geiss et al. 2008). Specifically, NanoString nCounter CodeSets were constructed for 27 *N*-glycosylation-related genes and three housekeeping genes, as previously described (Lee et al. 2014).

### Statistical analysis

All data are expressed as the mean ± standard deviation from triplicate experiments. Statistical analysis was performed using a two-tailed Student’s *t* test when appropriate. Differences between means were considered significant at *p* < 0.05.

## Results

### Effect of autophagy inhibitors on cell growth and Fc-fusion glycoprotein production

To examine the effect of autophagy inhibitors on cell growth and Fc-fusion glycoprotein production, we cultivated two rCHO cells, DUKX-Fc cells and DG44-Fc cells, producing the same Fc-fusion glycoprotein derived from DUKX-B11 and DG44, respectively. We selected three chemical autophagy inhibitors, namely SP600125, LY294002, and BafA1, which act at three different stages of autophagy.

**Fig. 1:**
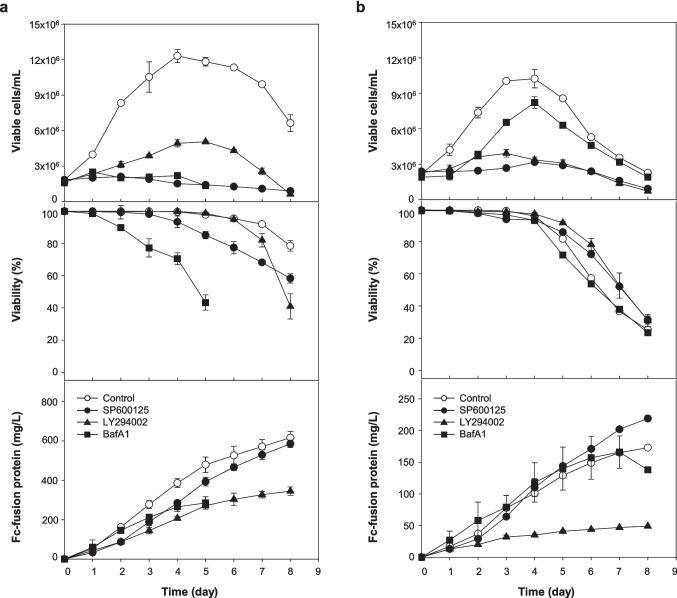
Profiles of cell growth, viability, and Fc-fusion glycoprotein production in two rCHO cell lines with and without three autophagy inhibitors. **a** DUKX-Fc cells and **b** DG44-Fc cells. Without autophagy inhibitor (open circle), with SP600125 (closed circle), with LY294002 (closed triangle), and with BafA1 (closed square). Error bars represent standard deviations based on triplicate experiments

Figure 1 shows the viable cell density, viability, and Fc-fusion glycoprotein concentration profiles of two cell lines during the cultures with or without autophagy inhibitors. In both cell lines, all autophagy inhibitors reduced cell growth and Fc-fusion glycoprotein production compared to the control culture. The vehicle control culture with the same amount of solvent (DMSO) did not exhibit any detrimental effects (*p* > 0.05) (data not shown). Although the extent of the effect varied due to differences in the timing of chemical treatment, concentration, initial seeding density, and cell culture medium, the results demonstrated similar trends to a previous study involving the same rCHO cells exposed to the same chemical inhibitors (Baek et al. 2016). Furthermore, we confirmed the accumulation of intracellular LC3-II protein, a key marker for measuring autophagic activity, through western blot analysis in both cell lines (Fig. S1). These findings confirmed that autophagy inhibition negatively impacts both cell growth and Fc-fusion glycoprotein production in rCHO cell culture.

### Effect of autophagy inhibitors on sialylation of Fc-fusion glycoprotein

To investigate whether autophagy inhibition affects the sialylation of Fc-fusion glycoprotein in rCHO cells, Fc-fusion glycoprotein produced in the cultures shown in Fig. 1 was purified by protein A chromatography. The isoform distribution, sialic acid content, and sialylated *N*-glycan proportion of the Fc-fusion glycoprotein were analyzed using IEF gel analysis, sialic acid content assay kit, and an anion exchange liquid chromatography, respectively. Except for the cultures with BafA1 on day 3 of DUKX-Fc cells, where cell viability decreased rapidly, supernatants from cultures with viability greater than 90% were harvested to avoid the effects of released sialidase resulting from cell membrane disruption.

**Fig. 2:**
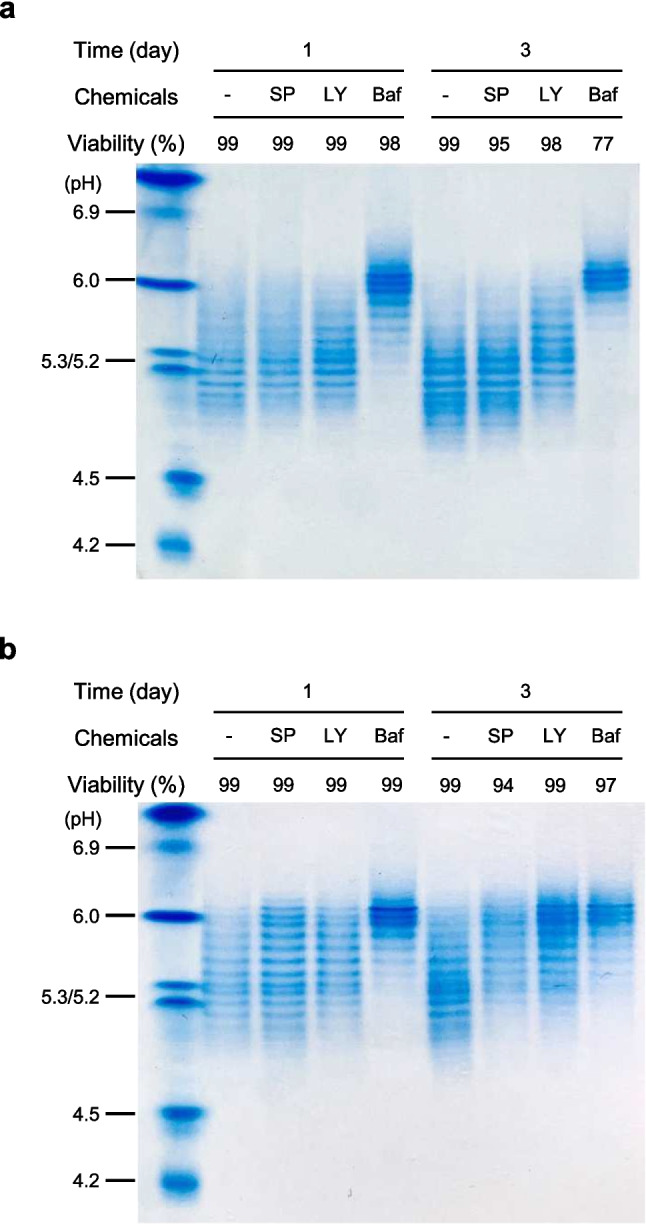
Isoelectric focusing (IEF) gel analysis of purified Fc-fusion glycoprotein produced in **a** DUKX-Fc cells and **b** DG44-Fc cells without autophagy inhibitor (−), with SP600125 (SP), with LY294002 (LY), and with BafA1 (Baf). Culture supernatants were harvested on days 1 and 3 of the cultures shown in Fig. 1 and purified by protein A affinity chromatography. IEF gel analysis was performed in triplicate experiments

Figure 2 shows the isoform distribution of Fc-fusion glycoprotein produced from DUKX-Fc cells and DG44-Fc cells treated with three autophagy inhibitors, as determined by IEF gel analysis. In both DUKX-Fc cells and DG44-Fc cells, the proportion of acidic isoforms of the Fc-fusion glycoprotein produced with autophagy inhibitors was lower than that without autophagy inhibitors. Among the three chemicals, the addition of BafA1 had the most significant detrimental effect on the sialylation of the Fc-fusion glycoprotein in both cell lines (*p* ≤ 0.05). For DUKX-Fc cells, the sialic acid content of the Fc-fusion glycoprotein in control culture on day 3 was 11.3 ± 0.1 mol sialic acid/mol protein. However, with the addition of SP600125, LY294002, and BafA1, the sialic acid content significantly reduced to 9.0 ± 0.1, 6.7 ± 0.2, and 1.5 ± 0.2 mol sialic acid/mol protein, respectively. A similar trend was observed in DG44-Fc cells: 6.1 ± 0.1, 3.8 ± 0.1, 2.6 ± 0.5, and 2.1 ± 0.2 mol sialic acid/mol protein for control, SP600125, LY294002, and BafA1, respectively.

**Fig. 3:**
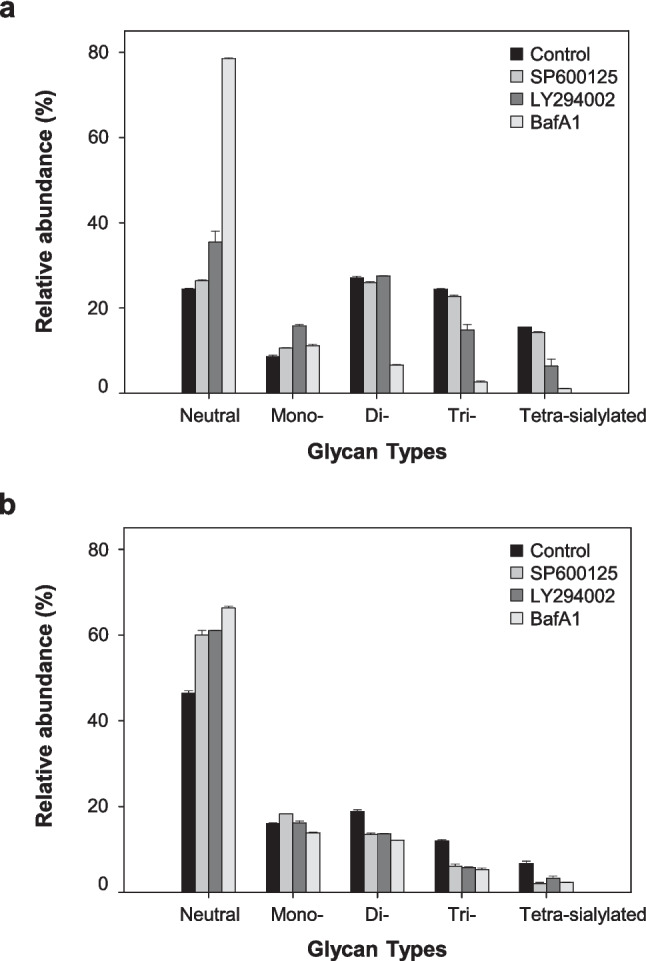
Anion exchange HPLC analysis of the sialylated *N*-linked glycans obtained from purified Fc-fusion glycoprotein in the absence and presence of the three autophagy inhibitors in **a** DUKX-Fc cells and **b** DG44-Fc cells. Relative abundance of neutral and sialylated *N*-linked glycans obtained from cultures without autophagy inhibitor (black), with SP600125 (gray), with LY294002 (dark gray), and with BafA1 (light gray). Error bars represent standard deviations calculated from triplicate experiments

Figure 3 shows the proportion of sialylated *N*-glycans categorized into five groups: neutral, mono-, di-, tri-, and tetra-sialylated. The proportion of sialylated *N*-glycans in DUKX-Fc cells and DG44-Fc cells treated with three autophagy inhibitors was analyzed using an anion exchange HPLC column and calculated through the integration of the areas within each group. In DUKX-Fc cells, the sum of the proportions of highly sialylated *N*-glycans (di-, tri-, and tetra-) in the control culture was 67.0% ± 0.2% on day 3. However, with the addition of SP600125, LY294002, and BafA1, this proportion decreased to 63.0% ± 0.2%, 48.7% ± 0.1%, and 10.3% ± 0.2%, respectively (*p* ≤ 0.05). Severe damage to sialylation was also observed in DG44-Fc cells. The sum of the proportions of highly sialylated *N*-glycan of Fc-fusion glycoprotein was 21.7% ± 0.3%, 22.8% ± 0.3%, and 19.8% ± 0.2% in SP600125, LY294002, and BafA1, respectively, which is lower than 1.5-fold compared to control culture (37.5% ± 0.4%) (*p* ≤ 0.05). Thus, the autophagy inhibition by chemical inhibitors significantly decreases the sialylation of Fc-fusion glycoprotein produced from rCHO cells.

### Effect of autophagy inhibitors on activities of sialidase and sialyltransferase

In the sialylation process of glycoproteins produced by mammalian cells, sialidase and sialyltransferase play major roles in removing or adding sialic acid to *N*-glycans. To elucidate the decreased sialylation resulting from autophagy inhibition, the activities of intracellular/extracellular sialidase and α2,3-ST were analyzed.

**Fig. 4:**
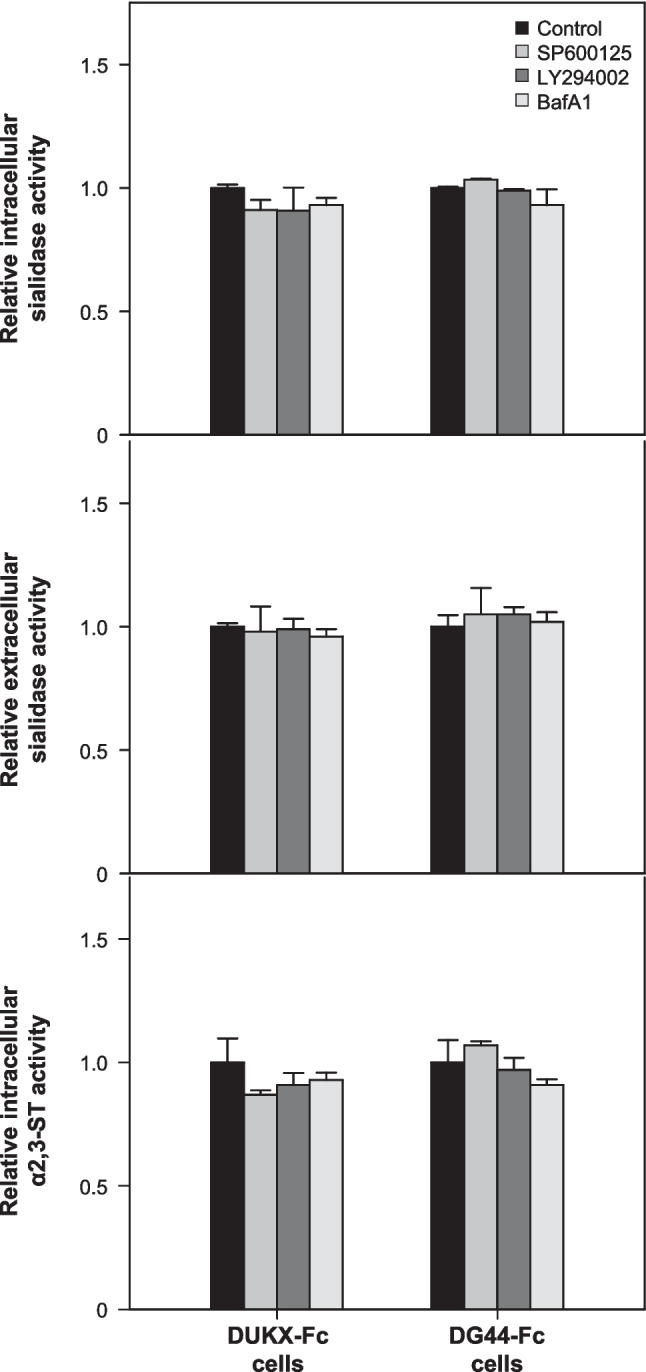
Relative activities of intracellular/extracellular sialidase and intracellular α2,3- sialyltransferase in the absence and presence of three autophagy inhibitors in DUKX-Fc cells and DG44-Fc cells. Without autophagy inhibitor (black), with SP600125 (gray), with LY294002 (dark gray), and with BafA1 (light gray). Cells and culture supernatants were taken on day 3 of the cultures shown in Fig. 1. Enzyme activity was normalized in the sample on day 3 of the culture without an autophagy inhibitor. Error bars represent standard deviations calculated from triplicate experiments

Figure 4 shows the relative activities of intracellular/extracellular sialidase and intracellular sialyltransferase in DUKX-Fc cells and DG44-Fc cells. The activities in cultures treated with autophagy inhibitors were normalized to day 3 of the control culture. In both cell lines, the activities of both intracellular and extracellular sialidase in all cultures treated with three inhibitors were not significantly different from those in the control culture (*p* > 0.05). Remarkably, even the culture on day 3 treated with BafA1 in DUKX-Fc cells, where the viability was below 90%, exhibited similar activities to the control culture. The relative activities of intracellular α2,3-ST also showed similar degrees in all cultures in both cell lines, regardless of the presence of autophagy inhibitors. These results indicate that the decrease in sialylation of the Fc-fusion glycoprotein did not result from increased sialidase activity or reduced sialyltransferase activity.

### Effect of autophagy inhibitors on intracellular nucleotide sugar pools

Nucleotide sugars in mammalian cells are crucial for the *N*-glycosylation process, and their availability can significantly impact the complexity of glycan and the final glycosylation profile (Naik et al. 2018). To determine whether autophagy inhibition influences the availability of nucleotide sugar, we quantified the amounts of intracellular nucleotide sugar pools, particularly focusing on the five most common nucleotide sugars found in CHO cells, using ion-pair reversed-phase HPLC.

**Fig. 5:**
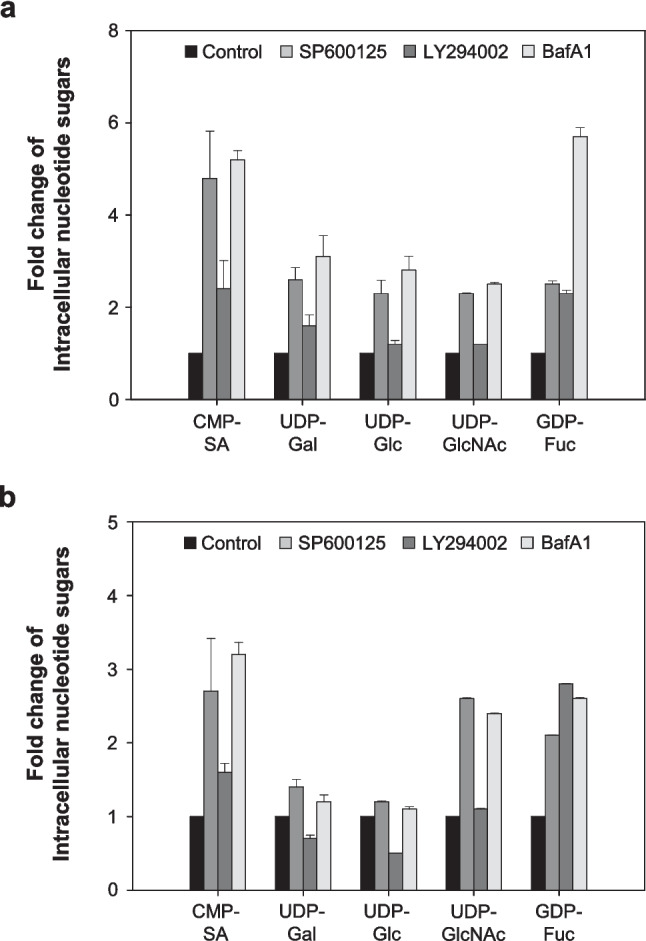
Fold changes of intracellular nucleotide sugar in the absence and presence of the three autophagy inhibitors in **a** DUKX-Fc cells and **b** DG44-Fc cells. Without autophagy inhibitor (black), with SP600125 (gray), with LY294002 (dark gray), and with BafA1 (light gray). Cells were taken on day 1 of the cultures shown in Fig. 1, and extracted nucleotide sugars were quantified by using ion-pair reversed-phase HPLC. Error bars represent standard deviations calculated from triplicate experiments

Figure 5 shows the relative amounts of five intracellular nucleotide sugars (CMP-SA, UDP-Gal, UDP-Glc, UDP-GlcNAc, and GDP-Fuc) for two rCHO cells in the presence and absence of autophagy inhibitors on day 1. In DUKX-Fc cells, all five nucleotide sugars remained highly abundant in cultures treated with the three autophagy inhibitors compared to the control culture. These levels were increased by more than 2.0-fold, particularly in cultures treated with SP600125 and BafA1 (*p* ≤ 0.05). CMP-SA, a nucleotide sugar directly involved in the final step of the sialylation process, was present at 4.8-fold, 2.4-fold, and 5.2-fold higher levels in cultures treated with SP600125, LY294002, and BafA1, respectively, compared to the control culture. A similar trend was also observed in DG44-Fc cells, although to a different extent. These results suggest that the inhibition of autophagy is likely to decrease sialylation by disrupting the availability of nucleotide sugars in rCHO cells.

### Effect of autophagy inhibitors on *N*-glycosylation-related gene expression

To investigate the reduced availability of intracellular nucleotide sugars by autophagy inhibition, the mRNA expression levels of *N*-glycosylation-related genes in both cell lines were analyzed through a NanoString nCounter system. A list of *N*-glycosylation-related genes was chosen based on our previous studies dealing with a NanoString nCounter system (Ha et al. 2015; Lee et al. 2017; Lee et al. 2014). Total RNAs were isolated from day 1 to day 3 of cultures with two rCHO cells and then analyzed in technical duplicates. Genes exhibiting an increase or decrease of more than 1.5-fold or having a *p* value less than 0.05 were considered differentially expressed.

**Fig. 6:**
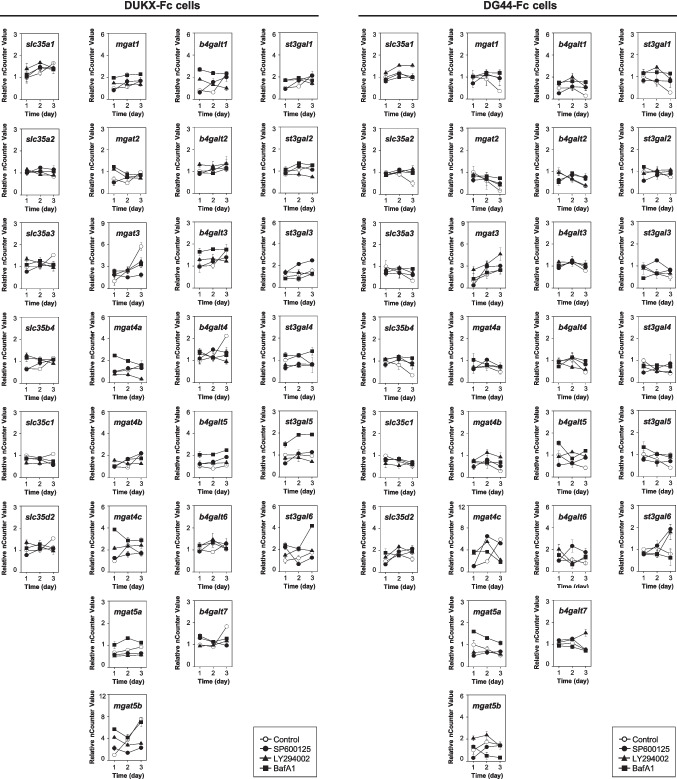
mRNA expression profiles of 27 *N*-glycosylation-related genes using a NanoString nCounter system in DUKX-Fc cells and DG44-Fc cells. Without autophagy inhibitor (open circle), with SP600125 (closed circle), with LY294002 (closed triangle), and with BafA1 (closed square). The relative nCounter value was normalized to the sample on day 1 of the culture without an autophagy inhibitor. Error bars represent standard deviations calculated from technical duplicates

Figure 6 shows the mRNA expression profiles of 27 *N*-glycosylation-related genes involved in the transport of nucleotide sugars to the Golgi apparatus and the transfer of saccharide moieties from nucleotides sugars to the *N*-glycan in Golgi. These 27 genes were classified into four groups based on their function: Six nucleotide sugar transporters, eight *N*-acetylglucosaminyltransferases, seven galactosyltransferases, and six sialyltransferases. In both cell lines, there was no statistically significant difference in the mRNA expression levels of the selected genes between the control culture and the cultures treated with the three chemical inhibitors (*p* > 0.05). Although the expression profile of some genes was significantly altered under certain conditions, they did not meet our criteria for being considered differentially expressed genes in both cell lines treated with the three chemicals. These results suggest that the limited availability of nucleotide sugars was not caused by altered expression levels of *N*-glycosylation-related genes.

### Effect of nucleotide sugar precursors feeding on sialylation of Fc-fusion glycoprotein under autophagy inhibition

To determine whether the reduced availability of nucleotide sugars under autophagy inhibition was associated with reduced activity of related enzymes, we supplemented the five nucleotide sugar precursors, ManNAc, Gal, Glc, GlcN, and GlcNAc, which are known to increase the concentration of intracellular nucleotide sugar pools for sialylation in rCHO cells (Cha et al. 2018).

**Fig. 7:**
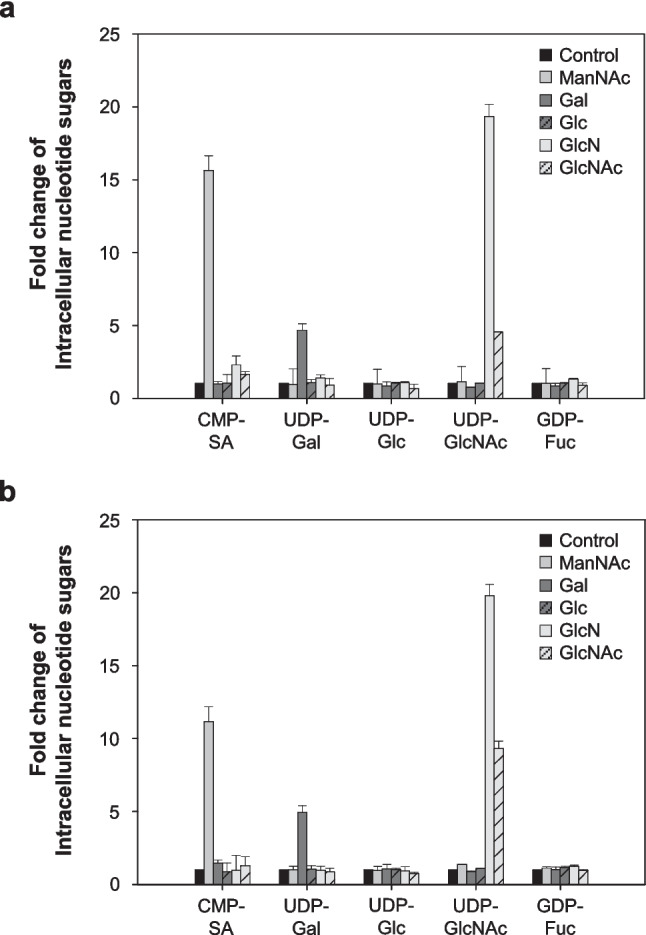
Fold changes of intracellular nucleotide sugar in **a** DUKX-Fc cells and **b** DG44-Fc cells supplemented with five nucleotide sugar precursors individually. Without nucleotide sugar precursor (black), ManNAc (gray), Gal (dark gray), Glc (dark gray striped), GlcN (light gray), and GlcNAc (light gray striped). Cells were taken on day 1 of the cultures shown in Fig. S2 and analyzed by ion-pair reversed-phase HPLC. Error bars represent standard deviations calculated from triplicate experiments

Figure 7 shows the relative amounts of five intracellular nucleotide sugars (CMP-SA, UDP-Gal, UDP-Glc, UDP-GlcNAc, and GDP-Fuc) individually supplemented with five nucleotide sugar precursors in two rCHO cells. Supplementation of ManNAc, Gal, GlcN, and GlcNAc dramatically increased the amount of intracellular nucleotide sugars CMP-SA, UDP-Gal, and UDP-GlcNAc in both DUKX-Fc cells and DG44-Fc cells (*p* ≤ 0.05). GlcN supplementation resulted in an increase in the intracellular UDP-GlcNAc pool compared to GlcNAc but had a negative impact on cell growth and production in DUKX-Fc cells (Fig. S2). The significantly increased pool of UDP-Gal is expected to compensate for the low levels of UDP-Glc by converting intracellular UDP-Gal to UDP-Glc via UDP-galactose-4-epimerase. Fortunately, GDP-Fuc is not a critical intracellular nucleotide sugar for sialylation, as the position of core fucosylation in *N*-glycan does not affect the consecutive binding of monosaccharides for sialylation and can, therefore, be excluded. Hence, ManNAc, Gal, and GlcNAc were chosen as the mixture of nucleotide sugar precursors.

To further evaluate the effect of simultaneous increase in intracellular pools of CMP-SA, UDP-Gal, and UDP-GlcNAc on the sialylation of Fc-fusion glycoproteins under autophagy inhibition, we cultivated DUKX-Fc cells and DG44-Fc cells with three autophagy inhibitors in the absence and presence of the nucleotide sugar precursor mixture.

**Fig. 8:**
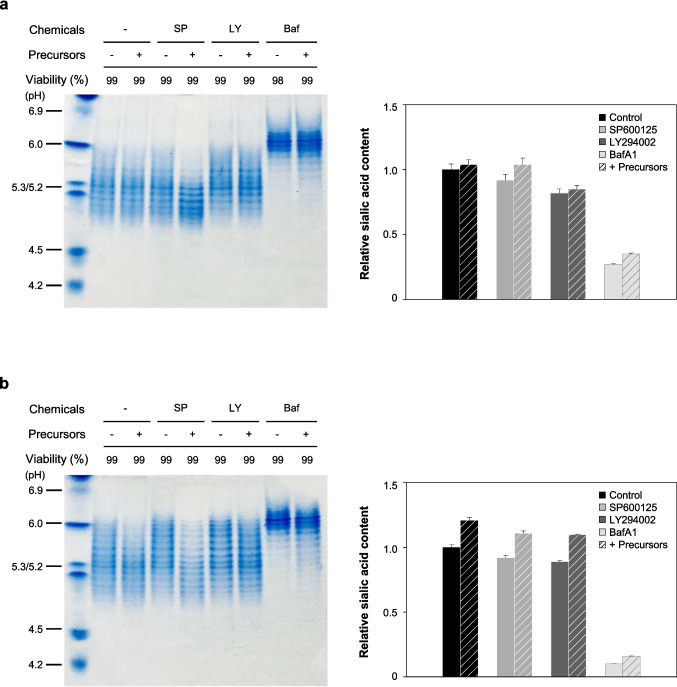
Sialylation patterns of the Fc-fusion glycoproteins obtained from the cultures with and without the mixed nucleotide sugar precursors under autophagy inhibition in **a** DUKX-Fc cells and **b** DG44-Fc cells. Isoelectric focusing (IEF) gel analysis of purified Fc-fusion glycoprotein produced in two rCHO cell lines without autophagy inhibitor (−), with SP600125 (SP), with LY294002 (LY), and with BafA1 (Baf). The relative sialic acid content of Fc-fusion glycoproteins produced from cultures without autophagy inhibitor (black), with SP600125 (gray), with LY294002 (dark gray), and with BafA1 (light gray). The supplementation of mixed nucleotide sugar precursors to control cultures and each inhibitor was indicated by a striped pattern coupled with each bar color. The relative sialic acid content was obtained by normalizing the sialic acid content of the Fc-fusion glycoprotein from culture without an autophagy inhibitor in the absence of the mixed nucleotide sugar precursors. Error bars represent standard deviations calculated from triplicate experiments

Figure 8 shows the isoform distribution and the sialic acid content of Fc-fusion glycoprotein from the two rCHO cell lines with the mixture of nucleotide sugar precursors under autophagy inhibition, analyzed by IEF gel analysis and sialic acid quantitation using UHPLC on day 1. The addition of mixed precursors to the cultures treated with inhibitors dramatically increased the intracellular nucleotide sugars, particularly CMP-SA, UDP-Gal, and UDP-GlcNAc, in both cell lines (data not shown). The proportion of acidic isoforms and the sialic acid content of Fc-fusion glycoprotein in DUKX-Fc cells and DG44-Fc cells were increased upon the addition of the nucleotide sugar precursor mixture, irrespective of autophagy inhibition. Interestingly, under autophagy inhibition by SP600125 and LY294002, cultures using a mixture of nucleotide sugar precursors could return to control culture levels at day 1. However, under the relatively severe stress conditions induced by BafA1 treatment, characterized by a high decrease in sialylation, the proportion of the acidic isoforms and the sialic acid content could not be restored to the levels observed in the control culture. Taken together, these results suggest that reduced sialylation of Fc-fusion glycoproteins under autophagy inhibition during rCHO cell culture disrupts the availability of intracellular nucleotide sugars, and excessive replenishment of nucleotide sugar precursors can restore sialylation by mitigating this stress environment in mildly stressful conditions but not under harsh condition.

## Discussion

In rCHO cell culture, autophagy occurs under various stressful culture conditions such as nutrient deprivation, hyperosmolality, and chemical additives (Han et al. 2010; Hwang and Lee 2008; Lee and Lee 2012a). When autophagy acts as a mechanism to promote survival under these stressful conditions by self-cannibalizing damaged organelles or cellular constituents, it can affect the production of therapeutic protein in rCHO cells by extending culture longevity and/or improving *q*_p_ (Baek et al. 2016; Lee and Lee 2012b). The occurrence of autophagic cell death, a type II PCD, at the end of batch culture can be harmful to quality of therapeutic glycoprotein due to release of enzymes from membrane-disrupted cells (Hwang and Lee 2008; Kim et al. 2009). Regardless of the survival- and death-promoting functions of autophagy, maintaining the sialylation of *N*-glycans within glycoprotein is an important factor in rCHO cell culture aiming to produce high-quality therapeutic glycoproteins. In this study, we demonstrated for the first time the effects of autophagy on the sialylation of Fc-fusion glycoproteins in rCHO cell culture.

The treatment of three chemical autophagy inhibitors leads to a decrease in the sialylation of Fc-fusion glycoprotein in two CHO cell lines (Figs. 2 and 3). Impairment of the sialylation of Fc-fusion glycoprotein was not caused by intracellular enzymes released due to cell membrane disruption, as evidenced by the high cell viability and low activity of extracellular sialidase (Figs. 1 and 4). It can be hypothesized that impaired glycoproteins were generated from rCHO cells due to unexpected changes in the internal environment, such as altered expression levels of *N*-glycosylation-related genes, concentrations of nucleotide sugar substrates, and activities of glycosyltransferases in the cells. The five intracellular nucleotide sugar pools remained highly abundant under autophagy inhibition in both rCHO cell lines, indicating that the availability of nucleotide sugars in the *N*-glycosylation process was limited (Fig. 5). The result of the NanoString nCounter assay with *N*-glycosylation-related genes suggests that the impaired availability of nucleotide sugar may be due to changes in the activity of proteins from these genes rather than mRNA expression levels. Interestingly, supplementation with nucleotide sugar precursors under mild stress conditions, characterized by low sialylation reduction, can restore the sialylation of the glycoprotein. However, this strategy was not found to be as effective under severe stress conditions, characterized by high sialylation reduction.

Among the three chemical inhibitors, the addition of BafA1 showed the most severe impairment of the sialylation of Fc-fusion glycoprotein in rCHO cells (Figs. 2 and 3). BafA1, a well-known vacuolar type proton-ATPase inhibitor, elevates lysosomal pH by inhibiting proton translocation and eventually blocks the fusion of autophagosomes with lysosomes (Yoshimori et al. 1991). In mammalian cells, BafA1 also increases Golgi pH and disrupts the acidification of Golgi by inhibiting proton translocation into the Golgi lumen (Yamamoto et al. 1998). Previously, it was reported that neutralizing Golgi pH by BafA1 addition induces the mislocalization of glycosyltransferases, such as *N*-acetylgalactosaminyltransferase, *N*-acetylglucosaminyltransfease, and galactosyltransferase, ultimately leading to the impairment of glycosylation in HeLa cells (Axelsson et al. 2001). Moreover, a 0.2 pH unit increase due to BafA1 treatment caused the impairment of α2,3-sialylation through mislocalization of α2,3-ST in COS-7 cells (Rivinoja et al. 2009). In CHO cells, more than 80% of galactosyltransferase and sialyltransferase activities are highest at pH 6.5, and BafA1 treatment significantly increased the pH values of the Golgi above pH 6.5 (Lee et al. 2019). Therefore, the severe impairment of sialylation caused by BafA1 treatment in this study may be partly due to intracellular pH changes. Thus, supplementation with nucleotide sugar precursors is expected to be unable to restore sialylation under autophagy inhibition by BafA1.

A decrease in sialylation of the Fc-fusion glycoprotein was also observed in two rCHO cell cultures treated with SP600125 and LY294002 (Figs. 2 and 3). While the mechanisms underlying the effects of these chemicals on *N*-glycosylation remain unclear, we noted a varying degree of decrease in sialylation across both cell lines. The introduction of LY294002 had a significant impact on the decrease of sialylation in DUKX-Fc cells compared to SP600125, whereas the addition of SP600125 and LY294002 resulted in a similar decrease in sialylation in DG44-Fc cells. Previous studies have highlighted significant genetic heterogeneity among cell lines due to extensive mutagenesis and clonal variation, although all CHO cell lines originated from a common ancestor (Lakshmanan et al. 2019; Reinhart et al. 2019). Lakshmanan et al. (2019) reported that this genetic heterogeneity among different CHO cell lines could influence the *N*-glycosylation profile of recombinant proteins. A glycomic analysis revealed notable differences in *N*- and O-glycosylation of host cell proteins produced from CHO-K1 cells, CHO-DXB11 cells, and CHO-DG44 cells. Interestingly, these significant differences in *N*-glycosylation occurred during processing in the Golgi and were not a result of gene copy number differences but rather the expression preferences of genes involved in glycan processing steps in each cell line (Lakshmanan et al. 2019). Therefore, the differences in the extent of the decrease in sialylation caused by autophagy inhibition in this study could be attributed to the genetic heterogeneity between the cell lines.

In CHO cells, nucleotide sugars, including CMP-SA, UDP-Gal, UDP-Glc, and UDP-GlcNAc, are essential for sialylated *N*-glycan formation (Tomiya et al. 2001). Generally, an increased concentration of nucleotide sugars in mammalian cells enhances the activity of glycosyltransferases, such as *N*-acetylglucosaminyltransfease and galactosyltransferase, which play an important role in *N*-glycan branching and galactosylation, with improved availability of substrates (Gramer et al. 2011). Our results showed that most intracellular nucleotide sugars required for sialylation remained at high levels under autophagy inhibition (Fig. 5). In this study, a decrease in the sialylation of Fc-fusion glycoproteins occurred under autophagy inhibition, despite seemingly increased concentrations of intracellular nucleotide sugars. Therefore, we speculate that the highly retained nucleotide sugars under autophagy inhibition are underutilized due to the decreased availability of *N*-glycosylation-related enzymes.

As important as nucleotide sugar availability, the expression and activity levels of glycosyltransferases, glycosidases, and transporters play an essential role in *N*-glycan synthesis (Naik et al. 2018). The decreased availability of nucleotide sugars due to autophagy inhibition may be influenced by altered expression levels of *N*-glycosylation-related genes involved in the transfer of nucleotide sugars to *N*-glycans in the Golgi apparatus. In this study, we assessed the expression levels of 27 *N*-glycosylataion-related genes using the NanoString nCounter system. In contrast to previous similar studies dealing with glycosylation-impaired culture conditions (Ha et al. 2015; Lee et al. 2017; Lee et al. 2014), autophagy inhibition did not significantly alter the mRNA expression levels of nucleotide sugar transporters or glycosyltransferases. Additionally, we analyzed the mRNA expression levels of 25 out of 52 *N*-glycosylation-related genes based on previous study (Lee et al. 2014) under autophagy inhibition, and we did not observe significant differences in the expressions of any of the genes in both cell lines (data not shown). Thus, the decreased sialylation of Fc-fusion glycoprotein by autophagy inhibition is likely to be a complex mechanism involving more than the *N*-glycosylation-related gene expressions, and further studies may consider elucidating the mechanism, including the activity of the *N*-glycosylation-related proteins.

Supplementation of the direct precursors required for nucleotide sugar biosynthesis, such as ManNAc, Gal, GlcNAc, uridine, and cytosine, has been shown to effectively restore decreased nucleotide sugar availability for *N*-glycan formation in glycoprotein (Del Val et al. 2016). As an example, Blondeel et al. (2015) reported that supplementing GlcNAc increased intracellular UDP-GlcNAc levels in CHO cell producing monoclonal antibody. The intracellular UDP-GlcNAc can be converted into ManNAc by UDP-GlcNAc 2-epimerase, contributing to an increased CMP-SA pool crucial for sialylation (Hinderlich et al. 2015). While GlcN is a preferable substance for elevating UDP-GlcNAc in CHO cells, it was excluded from supplementation due to its negative impact on cell growth and sialylation of glycoproteins in CHO cells (Blondeel et al. 2015; Lee et al. 2017). Wong et al. (2010) demonstrated that the supplementation of ManNAc and Gal notably increased the sialylation of interferon-γ up to 32% in CHO cells, emphasizing the importance of nucleotide sugar precursors for enhancing the sialylation. This strategy of supplementing nucleotide sugar precursors to elevate intracellular nucleotide sugar levels has proven effective in mitigating the reduced availability of nucleotide sugars, particularly in mild stress conditions induced by autophagy inhibitors.

Terminal sialylation is a critical concern for the quality of therapeutic glycoproteins, including tissue plasminogen activator and erythropoietin, as it significantly impacts *in vivo* half-life (Cole et al. 1993; Elliott et al. 2003). However, for canonical antibody production, our findings may not pose a major issue. Higher levels of sialylation in therapeutic monoclonal antibodies can interfere with binding to FcγRIIIa receptors, consequently reducing antibody-dependent cellular cytotoxicity (ADCC) (Scallon et al. 2007). Moreover, sialylation can mitigate the cytotoxicity of antibodies, particularly immunoglobulin G, without a concurrent increase in their half-life in serum, which is different from other therapeutic glycoproteins (Kaneko et al. 2006). Nevertheless, despite these characteristics of canonical antibodies, precise analysis of *N*-glycan profiles is imperative. Autophagy inhibition may affect not only sialylation but also the overall *N*-glycosylation profile. *N*-glycosylation in therapeutic antibodies is pivotal for efficacy, safety, and effector functions such as complement-dependent cytotoxicity and ADCC (Zhang et al. 2016). Therefore, a thorough and accurate examination of changes in *N*-glycosylation profiles under autophagy inhibition is essential, and techniques like liquid chromatography coupled with mass spectrometry are instrumental for this purpose.

The effect of autophagy induction on the sialylation of Fc-fusion glycoprotein in rCHO cells was also investigated using rapamycin, a well-known inducer of autophagy. In line with a previous study (Lee and Lee 2012b), autophagy induction enhanced the production of Fc-fusion glycoprotein in the same two rCHO cell lines used in the autophagy inhibition studies (Fig. S3). Interestingly, while autophagy inhibition negatively impacted the sialylation of the Fc-fusion glycoprotein, treatment with 200 nM rapamycin did not significantly alter the isoform distribution or proportion of the five sialylated *N*-glycan structures of the Fc-fusion glycoprotein during the early stages of batch culture, when cell viability was greater than 95% (Fig. S[Media MOESM1]). Furthermore, as anticipated, the improved cell viability observed at the end of batch culture due to rapamycin treatment had a positive impact on the sialylation of the Fc-fusion glycoprotein (data not shown). Given the diverse effects on sialylation observed upon autophagy inhibition with various chemicals, further investigation is warranted to explore the effectiveness of autophagy induction on the sialylation of Fc-fusion glycoproteins in rCHO cells using different autophagy inducers, including variations in the physical environment and chemical additives.

In conclusion, we demonstrated that autophagy inhibition negatively affects the sialyation of Fc-fusion glycoprotein in rCHO cell culture by disrupting the availability of intracellular nucleotide sugars. The supplementation of nucleotide sugar precursors can dramatically increase the amount of intracellular nucleotide sugars and restore the sialyation of Fc-fusion glycoprotein in mild stress conditions caused by autophagy inhibitors, but not in severe stress conditions.

## Supplementary information

ESM 1 (PDF 196 kb)

## Data Availability

The data that support for the findings of this study are all contained in the manuscript.
