# Supplementary material for: Effects of autophagy-inhibiting chemicals on sialylation of Fc-fusion glycoprotein in recombinant CHO cells
Source: Appl Microbiol Biotechnol. 2024 Feb 20;108:224. doi: 10.1007/s00253-024-13059-9 (PMC10879319; doi:10.1007/s00253-024-13059-9)
Supplement: Supplementary file 1 [file 253_2024_13059_MOESM1_ESM.pdf]

Applied Microbiology and Biotechnology

Supplementary data

Effects of autophagy-inhibiting chemicals on sialylation of Fc-fusion glycoprotein in recombinant CHO cells

Hoon-Min Lee[1,2], Jong-Ho Park[1,3], Tae-Ho Kim[1,4], Hyun-Seung Kim[1,2], Dae Eung Kim[5], Mi Kyeong Lee[5], Jungmok You[4], Gyun Min Lee[3], and Yeon-Gu Kim[1,2*]

Running title: Effect of autophagy inhibition on sialylation in rCHO cells

[1]*Biotherapeutics Translational Research Center, Korea Research Institute of Bioscience and Biotechnology (KRIBB), 125 Gwahak-ro, Yuseong-gu, Daejeon, Korea*

[2]*Department of Bioprocess Engineering, KRIBB School of Biotechnology, University of Science and Technology (UST), 217 Gajeong-ro, Yuseong-gu, Daejeon, Korea*

[3]*Department of Biological Sciences, KAIST, 335 Gwahak-ro, Yuseong-gu, Daejeon, Korea*

[4]*Department of Plant and Environmental New Resources, Graduate School of Biotechnology, College of Life Science, Kyung Hee University, 1732 Deogyeong-daero, Giheung-gu, Yongin-si, Gyeonggi-do, Korea*

[5]*College of Pharmacy, Chungbuk National University, Cheongju 28160, Korea*

** Correspondence to: Dr. Yeon-Gu Kim*

Tel: +82-42-860-4196; fax: +82-42-860-4149; e-mail: ygkim@kribb.re.kr

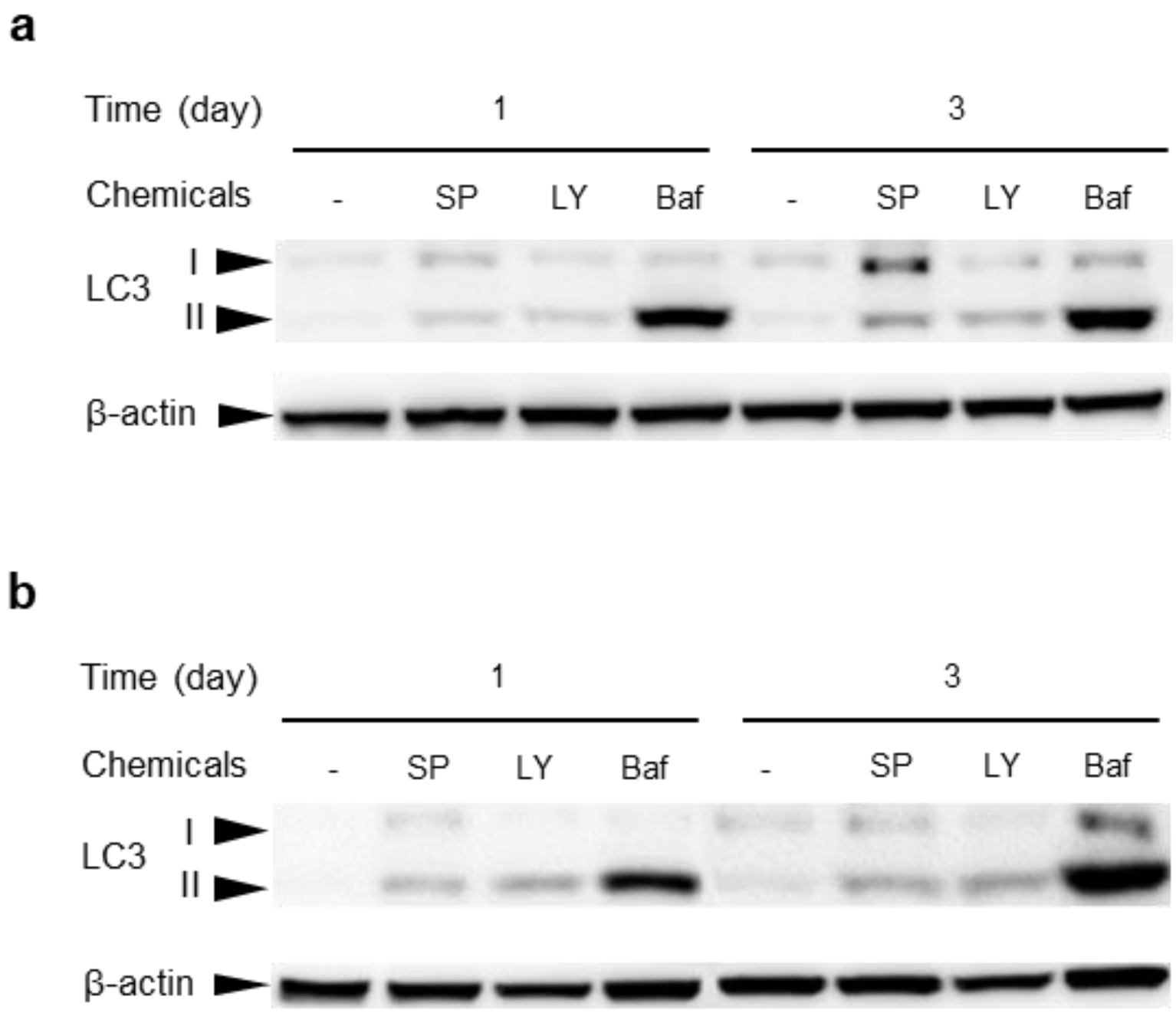

Fig. S1 Western blot analysis of the total cellular proteins from cultures in the absence and presence of three autophagy inhibitors in **a** DUKX-Fc cells and **b** DG44-Fc cells. Without autophagy inhibitor (-), with SP600125 (SP), with LY294002 (LY), and with BafA1 (Baf). An equal amount of cellular proteins from cell lysates at days 1 and 3 was separated on a 12% Bis-Tris NuPAGE gel.

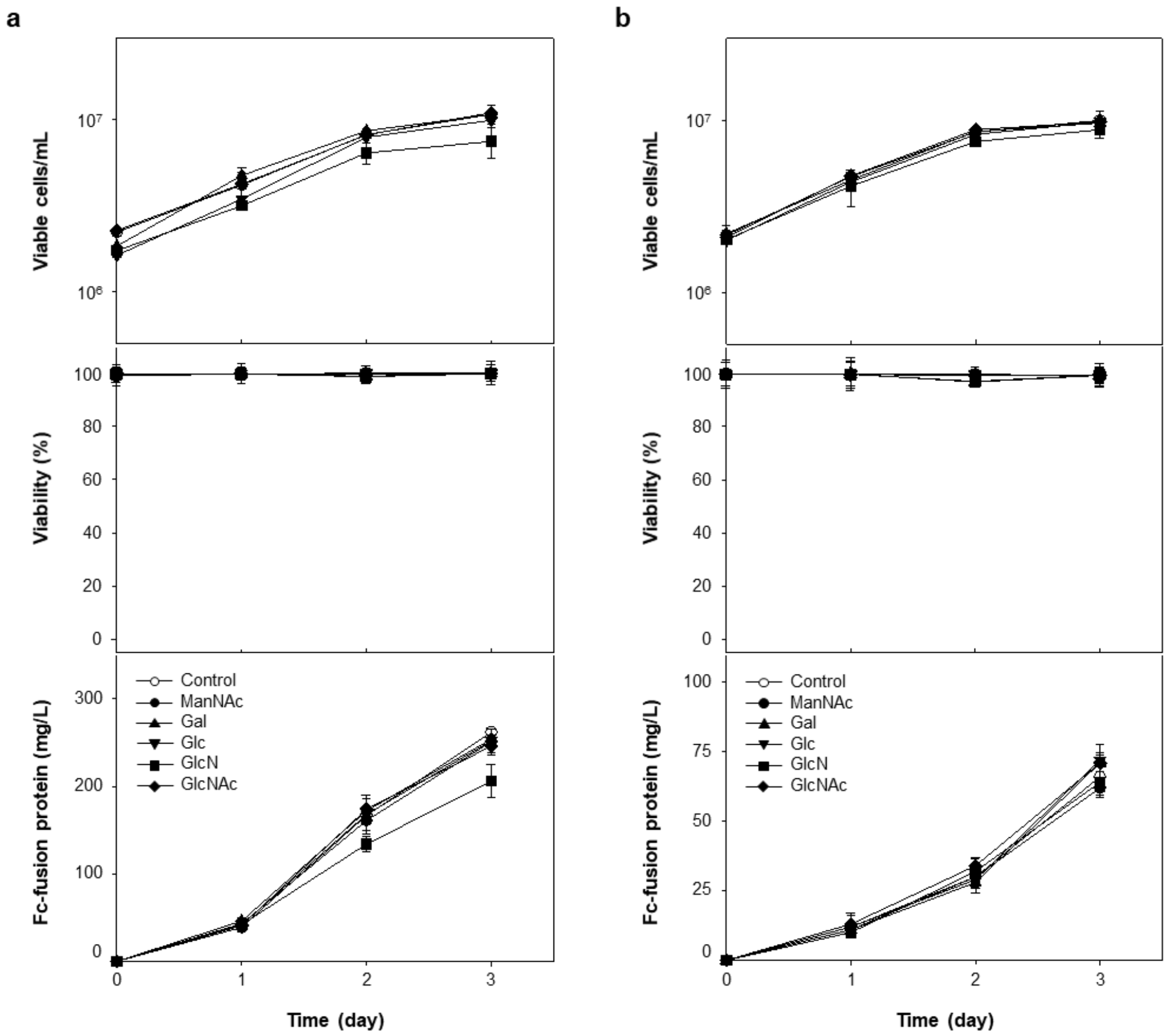

Fig. S2 Profiles of cell growth, viability, and Fc-fusion glycoprotein production of two rCHO cell lines individually supplemented with five nucleotide sugar precursors. **a** DUKX-Fc cells and **b** DG44-Fc cells. Without nucleotide sugar precursor (open circle), with ManNAc (closed circle), with Gal (closed up-triangle), with Glc (closed down-triangle), with GlcN (closed square), and with GlcNAc (closed diamond). Error bars represent the standard deviations of triplicate experiments

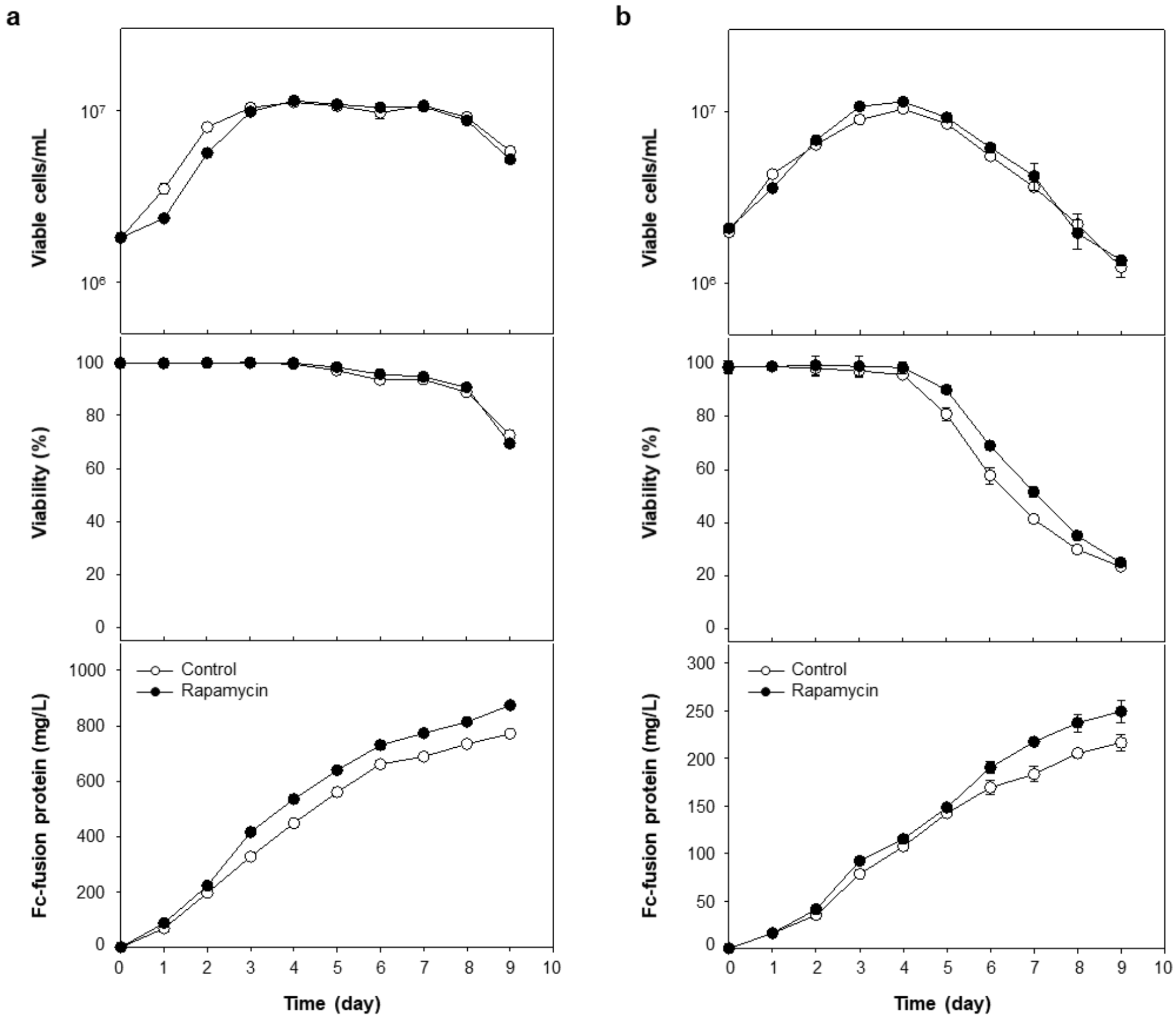

Fig. S3 Profiles of cell growth, viability, and Fc-fusion glycoprotein production of two rCHO cell lines in the absence and presence of rapamycin, a typical autophagy inducer. **a** DUKX-Fc cells and **b** DG44-Fc cells. Without rapamycin (open circle) and with 200 nM rapamycin (closed circle). Error bars represent the standard deviations of triplicate experiments

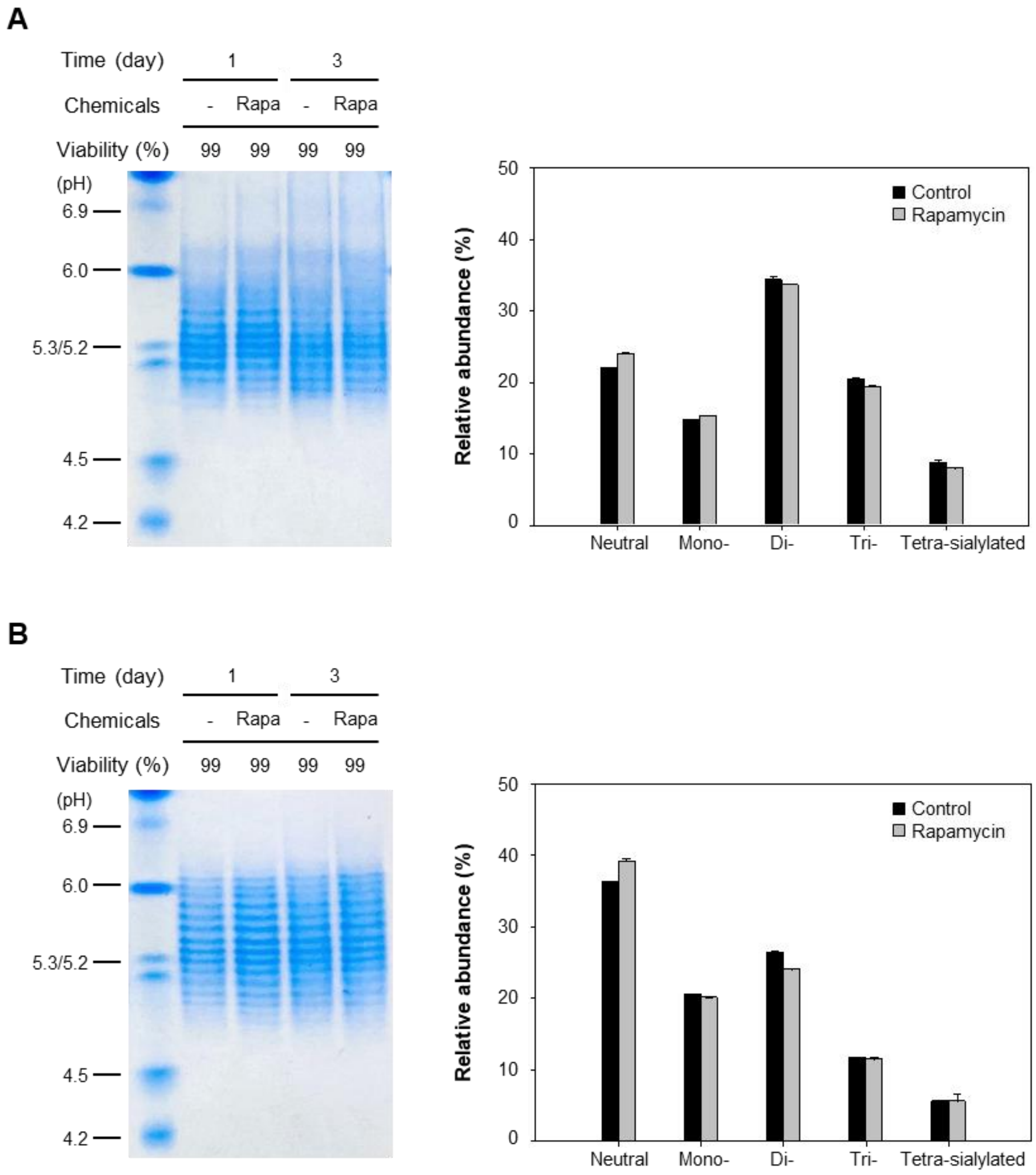

Fig. S4 Isoelectric focusing (IEF) gel analysis and sialylated *N*-glycan profiles obtained from purified Fc-fusion glycoprotein produced in the absence (- and black bar) and presence (Rapa and gray bar) of rapamycin in **a** DUKX-Fc cells and **b** DG44-Fc cells. Samples were taken on days 1 and 3 of the cultures, as shown in Fig. S2. IEF gel analysis was performed in triplicate experiments. Error bars in sialylated *N*-glycan analysis represent standard deviations calculated from triplicate experiments
